# Supplementary material for: A Review of the Health Benefits of Food Enriched with Kynurenic Acid
Source: Nutrients. 2022 Oct 8;14(19):4182. doi: 10.3390/nu14194182 (PMC9570704; doi:10.3390/nu14194182)
Supplement: Supplementary file 1 [file nutrients-14-04182-s001.zip › nutrients-1942370-supplementary.pdf]

Table S1. Content of kynurenic acid in human serum

Kynurenic acid content [μM]	Comment	Reference
Healthy adults		
0.071		[15]
0.06		[16]
0.045		[17]
0.045		[18]
0.038		[19]
0.032		[20]
0.027		[21]
0.023		[22]
0.017	pregnant	[23]
0.016	pregnant	[24]
Healthy children		
0.018		[25]

Table S2. Content of kynurenic acid in human cerebrospinal fluid

**Kynurenic acid content [μM]**	**Reference**
Healthy adults	
0.0017	[31]
0.00098	[32]
0.0009	[20]
0.0028-0.005	[33]
0.001	[34]
0.003 – 0.004	[21]
0.002	[16]
0.001	[35]
0.001	[36]
0.005	[37]
Children	
~0.005	[38]

Table S3. Excretion of kynurenic acid in human urine

Kynurenic acid content in urine	Reference	KYNA excretion [mg/day]*
Adults		
22.18 µM (male)<br>17.57 µM (female)	[47]	6.29 (male)<br>4.98 (female)
19.07 µM	[48]	5.41
18.96 µM	[49]	5.38
13.44 µM	[50]	3.81
13 µM	[51]	3.69
11.7 µM	[24]	3.32
4.69 µM	[52]	1.33
4.04 µM	[53]	1.14
15.9 µmol/day	[54]	3.01
11.04 µmol/day	[55]	2.09
9.97 µmol/day	[56]	1.88
9.3 µmol/day	[57]	1.76
0.74 µM mM^{-1} Cr	[58]	-
Children		
age under 14 years; mean age 5 years<br>93.12 µM (daytime)<br>74.07 µM (nighttime)	[59]	14.0-17.6
age 5-14 years<br>5.86 – 7.34 µg/mgCr	[60]	2.64 – 3.30
neonates<br>26.4 µg/mL x kg # (nocturnal)<br>42.2 µg/mL x kg # (diurnal)	[61]	-
mean age 37.7 or 44.0 months<br>5.5 µg/mL x kg # (nocturnal)<br>24.7 µg/mL x kg # (diurnal)	[62]	-

Note: *The assumption behind the calculations is that adult human excretes 1.5 liters of urine on a daily basis and child excretes 1 liter of urine on a daily basis. # The values obtained for each compound (in mg/100 ml urine) were multiplied by 10 and divided by the child's weight (in kg). Thus the results were expressed in mg/ml x kg.

Table S4. Content of kynurenic acid in food, beverages, herbs and spices for cooking

Food	Kynurenic acid content	Reference
Multiflorous honey	0.8773 μg/g	[73]
Bee pollen	0.6455 μg/g	
Broccoli	0.4184 μg/g	
Buckwheat honey	0.1812 μg/g	
Linden honey	0.1791 μg/g	
Potato	0.1301 μg/g	
Cauliflower	0.0473 μg/g	
Garlic	0.0276 μg/g	
Onion	0.0228 μg/g	
Milk 1.5%	0.0174 μg/g	
Yoghurt	0.0170 μg/g	
Corn	0.0160 μg/g	
Bread	0.0147 μg/g	
Kefir	0.0107 μg/g	
Barley	0.0098 μg/g	
Pig liver	0.0091 μg/g	
Pea	0.0088 μg/g	
Fluor	0.0084 μg/g	
Hard cheese	0.0084 μg/g	
Carrot	0.0073 μg/g	
Tomato	0.0059 μg/g	
Rice	0.0056 μg/g	
Egg	0.0046 μg/g	
Cucumber	0.0038 μg/g	
Pork	0.0037 μg/g	
Beef	0.0031 μg/g	
Sunflower oil	0.0027 μg/g	
Apple	0.0023 μg/g	
Colza oil	0.0021 μg/g	
Fish (roach)	0.0014 μg/g	
Red paprika	0.0012 μg/g	
Chestnut honey	up to 2114.9 μg/g	[74]
Eucalyptus honey	143.4 μg/g	
Honeydew honey	126.4 μg/g	
Sunflower honey	125.3 μg/g	
Acacia honey	23.1 μg/g	
Fungus *Suillus luteus*	0.0263 μg/g dry weight	[75]
Potato	up to 0.648 μg/g wet weight	[76]
French fries	up to 0.646 μg/g wet weight	
Crisps	up to 0.478 μg/g wet weight	
Potato flour	0.035 μg/g wet weight	
Wheat flour	0.008 μg/g wet weight	
Basil	14.08 μg/g	[77]
Thyme	8.87 μg/g	
Marjoram	3.78 μg/g	
Curry powder	3.39 μg/g	

Herbes de Provence	3.20 μg/g	
Mint	3.02 μg/g	
Oregano	2.50 μg/g	
Savory	2.37 μg/g	
Turmeric	1.48 μg/g	
Sage	1.28 μg/g	
Rosemary	1.21 μg/g	
Cloves	1.29 μg/g	
Tarragon	1.04 μg/g	
Glechoma	1.01 μg/g	
Bay leaf	0.91 μg/g	
Fennel	0.80 μg/g	
Parsley	0.76 μg/g	
Cumin	0.64 μg/g	
Black pepper	0.10 μg/g	
Chestnut honey	129 – 601 μg/g	[13]
Pine honey	14.2 μg/g	
Eucalyptus honey	11.3 μg/g	
Sunflower honey	1.73 μg/g	
Fir honey	1.06 μg/g	
Clover honey	0.342 - 0.746 μg/g	
Orange honey	0.609 μg/g	
Linden honey	0.391 μg/g	
Buckwheat honey	0.329 μg/g	
Orange honey	0.273 μg/g	
Sulla honey	0.222 μg/g	
Lavender honey	0.147 μg/g	
Thyme honey	0.143 μg/g	
Multiflorous honey	0.093 - 0.124 μg/g	
Honeydew honey	0.122 μg/g	
Lucerne honey	0.101 μg/g	
Tea	up to 0.087 μg/mL	[14]
Coffee	up to 0.006 μg/mL	
Cocoa powder	up to 4.486 μg/g	[78]
Yoghurt	up to 0.286 μg/mL	
Kefir	up to 0.242 μg/mL	
Red wine	up to 0.180 μg/mL	
White cheese	up to 0.077 μg/g	
Beer	up to 0.052 μg/mL	[11]
Mead	up to 0.381 μg/mL	
Wine	up to 0.109 μg/mL	
Beer	up to 0.052 μg/mL	
Distilled beverages	up to 0.016 μg/mL	
Wine	up to 0.065 μg/mL	[79]
Chestnut honey	410-1140 μg/g	[80]

References presented in the table are arranged chronologically according to the date of publication, so the name of the food product may appear more than once.

Table S5. Content of kynurenic acid in medicinal herbs and supplements

Herb	Kynurenic acid content	Reference
Peppermint leaf	3.82 µg/g dry weight	[83]
Nettle leaf	2.71 µg/g dry weight	
Birch leaf	2.68 µg/g dry weight	
Horsetail herb	2.27 µg/g dry weight	
Reumaflos tea	2.08 µg/g dry weight	
Elder flower	1.73 µg/g dry weight	
Reumaflex	1.66 µg/g dry weight	
Bean pericarp	0.57 µg/g dry weight	
Willow bark	0.26 µg/g dry weight	
Dandelion root	0.05 µg/g dry weight	
St. John's wort	5.06 µg/tablet	[82]
Broccoli	4.76 µg/tablet	
Lemonbalm	2.66 µg/tablet	
Small-leaved lime	2.26 µg/tablet	
Common nettle	2.03 µg/tablet	
Heartsease	0.99 µg/tablet	
Lupulin	0.55 µg/tablet	
Midland hawthorn	0.20 µg/tablet	
Flax	0.09 µg/tablet	
Chamomile	0.07 µg/tablet	

Table S6. Content of kynurenic acid in human milk and baby food

Food	Kynurenic acid content	Reference
Human milk	0.0257 μg/mL	[73]
Gerber – vegetables with veal	0.0148 μg/g	
BoboVita – vegetable soup	0.0056 μg/g	
BoboFrut – apple and carrot	0.0019 μg/mL	
Gerber-chicken	0.0010 μg/g	
Human milk	0.0108 μg/mL in the 7th day of breastfeeding; 0.0417 μg/mL in the 14th day of breastfeeding	[45]
Human milk	from 0.0039 μg/mL in the 3rd day to 0.0566 μg/mL in 6 months of breastfeeding	[46]
Milk baby formulas	up to 0.0015 μg/mL	

Table S7. Health-related effects of kynurenic acid (KYNA) administration

Effect/proerties	Species/tissue	KYNA treatment (dose, schedule)	Reference
Antiatherosclerotic	Mice, adult	5 mg/kg/day, intragastric; once a day for 8 weeks	[90]
	in vitro; BAEC line	0.1 mM	[108]
Antiinflammatory	Mice, female, adult	2.5, 5, 10 mg/kg; ip; acute	[109]
	Mice, male, adult	5 mg/kg/day; ip; for 1 month	[93]
	Mice, male and female, adult *C. albicans* intestinal infection	5 mg/kg, ip; day 3, 6, 9	[94]
	Rats, male, adult	300 mg/kg; ip; acute	[110]
	Rats, male, adult	100 mg/kg/h; iv; infusion for 6 h	[103]
	Rats, male, adult	25 mg/kg; iv; acute	[111]
	Dogs, adult	50 mg/kg; iv; infusion for 30 min in 20 mL	[102]
Antimigraine	Rats, male and female, adult	10, 50, 100 mg/kg ip; acute	[112]
	Rats, male, adult	189.17 mg/kg	[113]
	Rats, male, adult	300 mg/kg; ip; acute	[114]
	Rats, male, adult	1 mmol/kg (189 mg/kg); ip; acute	[115]
Antiobesity	Mice, adult	5 mg/kg/day, intragastric; once a day for 8 weeks	[90]
	Mice, male, adult	5 mg/kg/day; ip; for 1 month	[93]
Antioxidant	Rat brain homogenates in vitro	0.1–100 μM	[116]

	Rat brain homogenates and Xenopus laevis oocytes *in vitro*	0–150 μM	[116]
	Mice, adult	2.5, 25 or 250 mg/L of drinking water for different time periods 3, 7, 14, 28 days	[87]
Antiosteoporotic	Mice, female	5 mg/kg/day; ip; for 4 weeks	[97]
Antiulcerative	Mice, male, adult	300 mg/kg; ip; acute: once, or twice	[117]
	Rats, adult	unknown	[95]
Antiviral (SARS-CoV-2)	*in vitro*	IC_{50} = 0.58 μM	[118]
Bactericidal effect	*in vitro*	0.1, 0.5, 2.5 mM	[26]
Cardiovascular protection	Rat, adult aorta, ischemia-reperfusion model *in vitro*	0.1 mM	[119]
	Rabbit, adult heart, ischemia/reperfusion model *ex vivo*	20 mg delivered through aortic cannula over 30 sec	[120]
Cholesterol-lowering	Mice, adult	5 mg/kg/day, intragastric; once a day for 8 weeks	[90]
	Mice, male, adult	5 mg/kg/day; ip; for 1 month	[93]
Glucose tolerance improvement	Mice, male, adult	5 mg/kg/day; ip; for 1 month	[93]
Hepatoprotective	Rats, male, adult	500 mg/kg; ip; acute	[121]
	C57BL/6J male mice high-fat diet	2 mg/kg ip; injection every other day for 8 weeks	[122]
Protection against sepsis	Rats, male, adult	160 μmol/kg (30 mg/kg); ip; acute (2 injections)	[99]
	Rats, male, adult	160 μmol/kg (30 mg/kg); ip; acute (2 injections)	[100]

	Mice, adult	3x200 mg/kg; 500 mg/kg; sc; acute	[98]
Multi-organ dysfunction	Rats, adult	30-100 mg/kg; iv; 4 h before heat stress	[101]
Probiotic stimulating effect	*in vitro*	0.0005, 0.005, 0.05, 0.25, 0.5 mM	[123]
	Human, adults	0.5% cream; topical application	[106]
Wound healing	Rabbits; *in vitro*	0.5 mg/mL (cream; topical application); 0.00625-0.15 mg/mL (medium concentration)	[104]
	Rabbits	1% eye drops	[105]

Table S8. Clinical trials designed to investigate the kynurenic acid (KYNA) effect registered in ClinicalTrials.gov database

Study title	Goal / intervention	Study identifier
FS2 Safety and tolerability study in healthy volunteers	Aim of study is to evaluate the safety and tolerability of KYNA cream applied to the skin of healthy human subjects	NCT02340325
Pilot study investigating a restorative wound care cream together with petrolatum on surgical excisions	Objective of this pilot study is to investigate the safety and effectiveness of a new wound care product containing KYNA formulated to improve healing outcome, minimize complications of impaired healing and minimize the appearance of scars	NCT04169438
A clinical study investigating the effectiveness of OTC scar-management modalities	Phase II prospective, randomized, double-blind, placebo controlled and comparative clinical study evaluating hydrogel scar-management modalities for effective management of hyperproliferative scars and keloids	NCT04169490
Clinical safety and scar prevention study of a topical antifibrotic compound FS2	Study investigates the safety and effectiveness of daily post-surgical scar management, using a moisture-balancing base product containing different amounts of KYNA	NCT04186273

Table S9. Patents and patent applications claiming medical uses of kynurenic acid (KYNA)

Patent title	Claimed use of kynurenic acid	Patent identifier / priority date
Application of kynurenic acid in improving hyperlipidemia induced dyslipidemia, obesity and intestinal flora disorder	Application of KYNA in preparations for improving high-fat diet induced dyslipidemia, obesity and intestinal flora disorder	CN112168822A 2020-09-27
Composition for preventing or treating fatty liver disease comprising kynurenic acid	Pharmaceutical composition for the treatment or prevention of fatty liver disease comprising KYNA	KR20220005948A 2020-07-07
Methods for preventing and treating retinal damage	Method of treating, reducing the risk of, preventing, or alleviating at least one symptom of a retinal disease, injury or condition comprises a loss of retinal ganglion cells by administration of KYNA	US20210299114A1 2020-03-30
Compositions for use in the reduction of nociception and other health benefits in infants and adults	Nutritional composition promoting health benefits by increasing KYNA production by microbiota	WO2021116282A1 2019-12-11
Strontium based compositions and formulations for pain, pruritus, and inflammation	Composition containing strontium and KYNA for topical use for use in a method of treating chronic pruritus, pain, or inflammation	US20220054535A1 2018-12-21
Pharmaceutical composition based on kynurenic acid and its use in the prevention or reduction of overweight or obesity, and use of kynurenic acid	Pharmaceutical composition based on KYNA for the prevention of weight gain, overweight and obesity	PL427801A1 2018-11-16
Application of kynurenic acid or derivative thereof in preparing drug for improving pathological damages associated with chronic psychological stress	Use of KYNA for improving pathological injuries related to chronic mental stress	WO2020088270 A1 2018-10-29
New medical use of kynurenic acid and its salts in the treatment of the conjunctiva and cornea of the eye	Subject of the application is the medical use of KYNA, which inhibits the excessive growth of the conjunctival epithelium and the abnormal reconstruction of the eye conjunctiva	PL425606A1 2018-05-17
The use of kynurenic acid to reduce weight gain	Use of KYNA in subjects at risk of overweight and obesity, especially in the period of development and adolescence, for the prevention of overweight and obesity	PL239745B1 2017-01-17

The use of kynurenic acid and its salts in the treatment of diseases with an accelerated heart rate and the prevention of heart disease	Use of KYNA in the treatment of diseases with an accelerated heart rate and the prevention of heart diseases	PL234304B1 2016-12-07
Composition comprising kynurenic acid for relieving hangover	Composition containing KYNA for relieving hangover	KR101845704B1 2016-10-27
Use of kynurenic acid for treating muscle atrophy	KYNA for use as a medicament intended for increasing and/or maintaining muscle mass and/or power in a mammal	EP3506901B1 2016-09-05
Methods to reduce harmful effects of smoking cigarettes, cigars and tobacco, and of exposure to smoke from cigarettes, cigars and tobacco by using kynurenic acid in nanoparticle carrier systems	Method to reduce the harmful effects of smoking cigarettes, cigars, and tobacco, and exposure to smoke from the smoking cigarettes, cigars and tobacco characterized in that KYNA in a nanoparticle carrier system is added to the cigarettes, cigars or tobacco	WO2017217877A1 2016-06-15
Medical use of kynurenic acid and its salts for the treatment of urolithiasis	KYNA for use in the prevention or treatment of urolithiasis in the human and animal body	PL233551B1 2014-12-19
Kynurenic acid for the treatment of disturbances in the body's water and electrolyte balance	KYNA for use in the treatment of disorders of the body's water and electrolyte balance, preferably for the treatment and prophylaxis of diseases associated with excessive accumulation of fluid in the tissues	PL226845B1 2013-12-13
Anti-fibrogenic compounds, methods and uses thereof	Use of KYNA for treating fibrotic disease	US9737523B2 2013-06-05
The use of kynurenic acid in drug and medicine	Use of KYNA, characterized in that it is used in dietetics and medicaments, in particular in rheumatic diseases	PL226971B1 2009-05-15
Novel medical use of kynurenic acid, its precursors and derivatives	Use of KYNA in manufacturing of a drug used in prevention and therapy of pancreas disorders characterized by edema and/or vacuolization and/or destruction of pancreatic tissue and/or pancreas necrosis leading to release of pancreatic α-amylase	WO2010110689A1 2009-03-23
Use of kynurenic acid and derivatives thereof in the treatment of conditions of the gastrointestinal tract accompanied by hypermotility and inflammation or gout or multiple sclerosis	Use of KYNA in the manufacture of a medicament for the treatment of conditions characterized by pathophysiological conditions of the gastrointestinal tract accompanied by increased motility and an elevated activity of the enzyme xanthine oxidoreductase (XOR), and further for the treatment of gout or multiple sclerosis	WO2008087461B1 2007-01-17

Kynurenic acid and salts thereof for the prevention and treatment of shock	Use of KYNA for preparing drugs effective in the prevention and treatment of shock, in particular septic shock	WO2006117624A2 2005-04-29
Treatment of neurological diseases by increasing brain concentrations of kynurenic acid	Method for treating KYNA neurological disease in an individual comprising administering a metabolic precursor of kynurenic acid	US6265442B1 2001-07-24
EAA antagonists as anti-emetic drugs	Use of KYNA for suppressing emesis mediated by activation of excitatory amino acid receptors on the surfaces of neurons in brain regions accessible to blood-borne molecules that do not freely penetrate blood-brain barriers comprising administering to a susceptible mammal	US5039528A 1989-12-11
Kynurenic acid in neurodegenerative disorders	Use of KYNA for the preparation of medicament useful for the prevention or treatment of neurodegenerative disorders	IE882452L 1988-08-11

Table S10. Estimated daily intake of kynurenic acid as an ingredient of food

**Diet composition**		**Kynurenic acid daily intake [mg]**		
		**low**	**high**	**average***
Meat, vegetables, beverages	no honey	0.053	0.076	0.065
	with chestnut honey	0.325	41.3	20.81

*Average value was calculated as a mean of low intake and high intake values. Calculations presented are based on data derived from following Tables: S10-S13.

Table S11. Intake of kynurenic acid in vegetables

	Group of vegetables*				1 serving	Kynurenic acid** [µg]	
	green	yellow	cruciferous	other		low intake	high intake
Equally mixed portion [g]	20	20	20	20	80	4.2	14
Kynurenic acid content per 1 serving [µg]	0.04	0.09	1.25	0.03	1.4		
Green predominantly portion [g]	40	13	13	13	80	2.8	9.5
Kynurenic acid content per 1 serving [µg]	0.08	0.06	0.8	0.02	0.95		
Yellow predominantly portion (g)	13	40	13	13	80	3	10
Kynurenic acid content per 1 serving [µg]	0.02	0.18	0.8	0.02	1.01		
Cruciferous predominantly portion [g]	13	13	40	13	80	9.5	26
Kynurenic acid content per 1 serving [µg]	0.02	0.06	2.5	0.02	2.6		

*Allocation of vegetables in accordance with color classification of fruit and vegetables. Low intake was established as consumption of three or fewer servings per week. High intake response to upper quartile or quintile was established as consumption of more than 10 servings per week. The mean portion was established based on data describing specificity of meal composition declared in various countries [124]. **For all specific kinds of vegetables, the mean amount of KYNA was calculated based on our recent analysis [73].

Mean kynurenic acid content in various vegetables was calculated from previously published data (for references see Table S4). The predominant consumption of various vegetable types was suggested as consumption of about 50% more than calculated in equally mixed vegetable portion.

Table S12. Intake of kynurenic acid in meat, dairy, egg and beverages

Category	Food	Kynurenic acid content* [mg/g]	Daily intake [g or mL]	Daily intake of kynurenic acid [mg]
Meat	Fish (roach)	0.000001	150	0.0002
	Beef	0.000003	150	0.0005
	Pork	0.000004	150	0.0005
	Pig liver	0.000009	150	0.0014
Dairy	Milk 1.5%	0.00002	200	0.0035
	Kefir	0.00024	150	0.0363
	Yoghurt	0.00029	100	0.0287
	White cheese	0.00008	100	0.0077
	Hard cheese	0.00001	100	0.0008
Egg	Egg	0.000005	56	0.0003
Beverages	Tea	0.00009	500	0.0435
	Coffee	0.00001	150	0.0009
	Wine	0.00018	500	0.0359
	Beer	0.000005	200	0.0026

*highest reported in the literature (see for details and references Table S4).

Table S13. Intake of kynurenic acid in single and daily dose of honey

Honey	Single dose [g]	Daily dose [g]	Content of kynurenic acid in honey [μg/gram]	Intake of kynurenic acid in single dose [mg]	Intake of kynurenic acid in daily dose [mg]
Chestnut honey	20	60	550	11	33.00
Pine honey	20	60	14.2	0.28	0.85
Eucaliptus honey	20	60	11.3	0.23	0.68
Sunflower honey	20	60	1.73	0.03	0.10
Buckwheat honey	20	60	0.33	0.007	0.02
Chestnut honey		75	550		41.25
Pine honey		75	14.2		1.07
Eucalyptus honey		75	11.3		0.85
Sunflower honey		75	1.73		0.13
Buckwheat honey		75	0.33		0.02

Single dose and daily dose of honey according to: https://www.rxlist.com/honey/supplements.htm. Content of kynurenic acid in honey according to Turski et al., 2016 [13].

Table S14. Effects of chestnut honey established in animals, *in vitro* and biochemical studies

Subject	Treatment	Effect	Reference
Animal studies			
Four-week-old male C57BL/6J mice	HFD supplemented with Sicilian black bee chestnut honey for 16 weeks;<br>dose of honey:<br>45 mg honey ingested/day/mouse	• attenuation of obesity-related peripheral and central insulin resistance<br>• improvement of glucose metabolism in HFD mice<br>• mitigation of neurodegeneration induced by HFD<br>• prevention of increase in pro-apoptotic gene expressions, such as Fas-L and p-27, and decrease in anti-apoptotic genes, such as Bcl-2 and BDNF caused by HFD<br>• improvement of insulin signaling in brains of HFD mice<br>• modification of blood levels of leptin and adiponectin	[127]
Male rats HCl/ethanol-induced gastric ulcers	HCl/ethanol-induced gastric ulcers;<br>dose of honey:<br>1.25 and 2.5 g/kg;<br>orally for three consecutive days	• prevention<br>• ulcer preventive index<br>dose 1.25 g/kg – 11.4%<br>dose 2.5 g/kg – 77.1%	[128]
Adult female Sprague Dawley rats weighing 250–300 g	CCl_4 –induced liver damage;<br>dose of honey:<br>400 mg/kg;<br>gavage for 7 days	• enhanced healing of CCl4-induced liver damage	[129]
New Zealand White female rabbits	Four 1.5 cm x 1.5 cm full-thickness skin wounds were created on the back of each animal and treated with pure honey or sterile saline, respectively;<br>wounds were assessed by wound measurements and collection of samples at 7, 14, and 21 days post wounding to evaluate the healing process	• increased formation of granulation tissue, epithelization, angiogenesis, and fibroplasia levels increased in the honey-treated groups	[130]
Rats	Alkaline burn on cornea;<br>0.05% chestnut honey eye drops<br>four times a day for 14 days	• better corneal healing after treatment with chestnut honey containing eye drops concerning corneal healing sore and $\alpha4\beta1$ integrin staining	[131]
in vitro studies			
Breast cancer, MCF7, SKBR3, MDAMB-231,	*in vitro*<br>concentration of honey:<br>1, 2.5, 5, 7.5, and 10 mg/mL	• chestnut honey induced cytotoxicity over 50% on all the cell lines, including the control	[132]

fibrocystic breast epithelial cell line; MCF10A as control		MCF10A cells, even with low doses starting from 1 mg/mL<br>• chestnut honey displayed remarkably cytotoxic effect on breast cancer cell lines, MCF7, SKBR3, and even on the most aggressive MDAMB 231, representing the triple negative breast cancer, which lacks of targeted anticancer therapy	
Enzyme isolated from rat liver microsomes	*in vitro*<br>effective IC_{50} concentration: 41.60 μg/mL	• inhibition of monoamine oxidase (MAO)	[133]
Urease and xanthine oxidase activity, antioxidant capacities determined using total phenolic content, ferric reducing antioxidant power (FRAP), and DPPH radical scavenging activity assays		• xanthine oxidase IC_{50}: 0.028–0.039 g/mL<br>• urease IC_{50}: 0.010–0.034 g/mL<br>• total phenolics IC_{50}: 38.9–65.3 mg GAE/100 g sample<br>• FRAP IC_{50}: 3.11-4.69 μmol $FeSO_4$ x7H_2O/g sample<br>• DPPH-SC_{50}: 35.1-41.35 mg/mL	[134]
Staphylococcus aureus and *Escherichia coli*	*in vitro*	antibacterial properties	[135]
Escherichia coli, Pseudomonas aeruginosa, Enterococcus faecalis, Staphylococcus aureus	*in vitro*<br>monofloral Etna *Castanea sativa Miller* honeys	• antioxidant, NO scavengers, DPPH, FRAP and antimicrobial properties;<br>• high antioxidant capacity against free radical species, including reactive oxygen species and peroxynitrite radical;<br>• DPPH and FRAP values confirm good antioxidant activity of chestnut honey	[136]
Biochemical studies			
Analytical assays	antioxidant and cytotoxic properties	utility of honey addition to potentiate the antioxidant and cytoprotective properties of medicinal plant based infusions	[137]
Analytical study	antihyaluronidase, antiurease, antioxidant, antimicrobial and physicochemical properties	comparative study	[138]

Analytical study	physico-chemical properties, antioxidant capacities and phenolic profiles	comparative study	[139]
Analytical study	physico-chemical properties	comparative study	[140]
Analytical study	nutritional value and antioxidant activity of honeys	comparative study	[141]
